# Successful Tracheotomy

**Published:** 1837

**Authors:** 


### Art. VII.—Successful Tracheotomy.

Dr. William Mount, of the vicinity of Cincinnati, has lately performed this operation with entire success, for the extraction of a foreign body from the trachea. His patient was a girl six years of age. She was eating unburnt coffee, or had the grains in her mouth, at the moment, when in play, she was thrown from a low porch. A violent, suffocative cough instantly ensued, and lasted without abatement for half an hour; a kind of intermission then occurred, but was followed by renewed paroxysms of cough, in each of which the patient was nearly strangled. Her parents administered several emetics, without effect. At the end of five weeks, Dr. Mount was called in. The patient was emaciated, and, to a considerable degree, hectical. Every forenoon, she was chilly; in the afternoon and early night, had fever with flushed cheeks; in the morning a copious perspiration. Her cough was hoarse and spasmodic, with now and then some expectoration. By the stethoscope, he discovered that the right lung was nearly silent—the left affected with mucous rattle.

Believing the death of the child inevitable without the removal of the foreign body, which both he and the parents supposed to have fallen through the rima of the glottis, the Doctor proposed tracheotomy, although the state of the lungs and the general condition of the patient's system, seemed almost to forbid the use of the knife. The parents consenting, he proceeded to the operation. The incision was made through the cricoid cartilage and first ring of the trachea, without wounding any branch of the thyroid artery. Not an ounce of blood was lost. Almost immediately, three fragments of a coffee grain were expelled by coughing. A probe was then passed down to the bifurcation of the trachea, and afterwards a pair of curved forceps; but nothing was felt. Light dressings of charpie were then applied, and an anodyne draught administered. In the course of the night following, she had several severe fits of coughing. The next day, on removing the lint, there was found upon it a larger fragment, amounting to nearly half a grain. The breathing was now easier; there was still a mocous rattle in the left lung, but the respiratory murmur had returned in the right. The cough and fever now abated rapidly, and in a week she was running about the house. The wound healed up kindly, and at the present time, three months after the operation, the patient is entirely restored. D.

